# Optimization of the Management of Category III Thyroid Nodules Using Repeat FNA and TIRADS

**DOI:** 10.3390/cancers14184489

**Published:** 2022-09-16

**Authors:** Dorota Słowińska-Klencka, Mariusz Klencki, Joanna Duda-Szymańska, Bożena Popowicz

**Affiliations:** 1 Department of Morphometry of Endocrine Glands, Medical University of Lodz, 251 Pomorska Str., 92-213 Lodz, Poland; 2 Department of Pathomorphology, Medical University of Lodz, 251 Pomorska Str., 92-213 Lodz, Poland

**Keywords:** thyroid, cancer, FNA, Bethesda system, AUS, FLUS, nuclear atypia, architectural atypia, EU-TIRADS

## Abstract

**Simple Summary:**

Thyroid nodules of category III in the Bethesda system do not constitute a uniform group, so it is still a challenge to produce a recommendation for the management of patients with such nodules. The aim of the study was to examine the benefits of the joint use of repeat FNA and a sonographic risk stratification system in category III nodules in relation to the kind of atypia: cytologic/nuclear vs. architectural. Our results indicate that in both kinds of these nodules, the joint evaluation of repeat FNA outcome and sonographic risk of malignancy shows better effectiveness than any of these methods separately. Such a combination allows the identification of patients with a high risk of malignancy as well as a group of patients in which surgical treatment may be safely put aside.

**Abstract:**

The aim of the study was to examine the benefits of the joint use of repeat FNA (rFNA) and EU-TIRADS in category III nodules in relation to the kind of atypia: nuclear vs. architectural (denoted by AUS and FLUS respectively). The study included 127 AUS and 1739 FLUS nodules with a known category of EU-TIRADS. Repeat FNA was performed in 82 AUS and 934 FLUS nodules of which 57 and 515 were excised, respectively. AUS nodules had higher malignancy risk than FLUS nodules. EU-TIRADS showed higher accuracy for AUS nodules, the opposite to rFNA, that had higher accuracy for FLUS nodules. The combined criterion for AUS nodules (at least rFNA-V or EU-TIRADS-4) maximized sensitivity (92.3%) with acceptable specificity (70.0%); OR: 28.0. In the case of FLUS nodules, the combined criterion (rFNA-V or EU-TIRADS-5) maximized specificity (95.2%) with 57.7% sensitivity and a low percentage (13.9%) of positive nodules, OR: 27.0. In both types of nodules, the low risk category in EU-TIRADS and benign result of rFNA excluded cancer. Concluding, category III nodules with and without nuclear atypia differ in their risk of malignancy and, consequently, diagnostic criteria adopted for the evaluation of these nodules with rFNA and EU-TIRADS should be specific to AUS and FLUS nodules.

## 1. Introduction

Fine needle aspiration biopsy (FNA) combined with ultrasound imaging (US) is a standard method used for the assessment of thyroid nodules. Unfortunately, the method has some inherent problems, of which the most prominent one is the relatively high frequency (reaching 30%) of equivocal outcomes of the cytological examination. Among those equivocal results, the outcomes especially difficult to interpret are these of category III in the Bethesda System for Reporting Thyroid Cytology (BSRTC): follicular lesion of undetermined significance—FLUS/atypia of undetermined significance—AUS [1,2]. That category was established in 2008 and since then no general agreement on how it should be formulated and interpreted has been reached. The authors of the BSRTC recommended the use of both aforementioned terms (AUS and FLUS) while assuming they should be regarded as synonyms for the sake of clinical interpretation. Category III was intended to constitute no more than 7% of FNA results, and the associated risk of malignancy (RoM) was meant to be under 15% [1]. Consequently, the regular management was to consist of repeat FNA with the consideration of molecular testing if possible. Numerous reports questioned those assumptions: it was found that the frequency of category III was significantly higher in some centers and its associated RoM differed according to the nature of the atypia, exceeding 40% or even 80% in some centers, particularly in the case of nodules with nuclear/cytologic atypia, commonly referred to as AUS [2,3,4,5,6,7]. The percentage of nodules showing this kind of atypia is particularly high in iodine-rich areas, where papillary cancer (PTC) decidedly predominates among other thyroid cancers. On the other hand, in iodine-deficient or postendemic areas, the nodules with architectural atypia, frequently described as FLUS, are the most common among category III diagnoses. In addition, on histopathological examination, these nodules are frequently found to be hyperplastic nodules or follicular neoplasms (both adenomas and carcinomas) [3,6,7]. Consequently, RoM in nodules with architectural atypia is reported to be even twice as high as in nodules with nuclear/cytologic atypia in many centers, including ours [4,5,7,8,9].

The diverse profiles of lesions corresponding to AUS and FLUS nodules result in a variable efficiency of the repeat FNA (rFNA) and sonographic risk-stratification systems (SRSs) used for the evaluation of these nodules. We examined that problem and showed that in patients with a repeated outcome of category III due to the architectural atypia without signs of nuclear/cytologic atypia, RoM did not increase in comparison to patients with a single diagnosis of FLUS (and remained in the range 3.2–13.0%) [10]. However, in patients with category III diagnosed twice and AUS identified at least once, RoM was significantly higher: 16.7–50.0% and prompting surgical treatment. We also analyzed the diagnostic efficiency of SRSs in nodules with equivocal cytology and we showed that it decreased along with decreasing percentage of PTC among cancers [11]. Other studies confirmed that observation [12]. It is not surprising as SRSs have been optimized to reveal the most common thyroid cancers (i.e., PTC) and are less efficient in the case of other cancers, particularly follicular thyroid carcinoma (FTC). Previously, we had not analyzed the efficiency of rFNA and SRS combined for the evaluation of category III nodules in relation to the kind of observed atypia. No similar study had been reported, thus we decided to examine the possible benefits of the joint assessment of rFNA category and the category of sonographic image of the nodule for both types of category III nodules. Our earlier comparative analysis of six SRSs showed that the system recommended by the European Thyroid Association (EU-TIRADS) had the greatest versatility in relation to various types of cancers [11]. That is why we decided to use that SRS.

## 2. Materials and Methods

### 2.1. Patients

FNA and US examinations were performed in a single center, in the years 2010–2021, of patients referred by endocrinologists from outpatient clinics. Over that period, 1946 nodules revealed in 1901 patients were classified into category III of BSRTC. Features of cytologic/nuclear atypia were identified in 134 of those nodules (that subcategory was denoted by the term AUS). Features of architectural atypia that were not accompanied by cytologic atypia were identified in 1784 of category III nodules (and such nodules were denoted by FLUS). The remaining 23 cases were placed in category III because of a compromised specimen (e.g., low cellularity, poor fixation, obscuring blood). Then, all nodules satisfying any of the following criteria were excluded from the analysis: (i) nodules in patients with a history of surgical or radioiodine thyroid treatment or neck irradiation, (ii) nodules coexisting with another nodule classified into category V or VI of BSRTC, (iii) nodules without full ultrasound imaging data (see Figure S1 in Supplementary Materials). Repeat FNA were performed in 80 patients with 82 AUS nodules and in 926 patients with 934 FLUS nodules. If several repeat FNAs were performed on a patient, the results of first rFNA were considered or the results of the second rFNA performed not later than 6 months after the first rFNA that had been non-diagnostic (category I of BSRTC). Surgical treatment directly after first FNA led to the excision of 34 AUS nodules and 377 FLUS nodules, while 23 AUS nodules and 151 FLUS nodules were excised after rFNA. Altogether, there were 572 patients treated surgically (57 with AUS nodules and 515 with FLUS nodules). The clinical decision on whether to perform rFNA or to refer the patient directly to surgical treatment was made by a physician in the outpatient clinic and was never influenced by our study design. Cancers were diagnosed in 94 of the excised nodules, more specifically in 23 AUS nodules and 71 FLUS nodules (Table S1). The incidence of PTC in AUS malignant nodules was higher than in FLUS malignant nodules: 87.0% vs. 49.3% (*p* = 0.0033). Table 1 shows demographic data on patients with AUS and FLUS nodules.

### 2.2. Microscopic Examination

FNAs were performed following regular procedures on thyroid nodules with a diameter of at least 5 mm (and usually over 1 cm), according to the recommendations in effect in our country [13,14]. In all cases, two aspirations of a nodule were carried out. Smears were immediately fixed with 95% ethanol solution and stained with hematoxylin and eosin. The results of FNA were formulated by pathologists with over ten years’ experience in thyroid pathology. BSRTC classification in the version prior to the modification in 2017 was applied [1,2]. According to this version, cases that demonstrated the nuclear features of PTC were excluded from category IV: suspicious for a follicular neoplasm. The diagnosis of FLUS was made when the specimen showed features from the borders of categories II and IV, especially some architectural atypia (microfollicles, trabeculae, or crowding), but in a degree insufficient for the diagnosis of neoplasia. The diagnosis of AUS was made when local features suggestive of PTC (nuclear grooves, enlarged nuclei with pale chromatin and alterations in nuclear contour and shape) were present in an aspirate that was otherwise benign in microscopic appearance or for specimens with limited cellularity but with nuclear atypia. When features typical of FLUS and AUS coexisted in a nodule, it was classified as an AUS nodule. A detailed description of the classification of nodules into specific diagnostic categories of the Bethesda system, as well as the risk of malignancy related to particular categories at our center were presented in our earlier report [15]. All the patients gave their informed consent to perform FNA.

The histopathologic examination was performed according to the standard procedure and its results were formulated according to the WHO classification of thyroid tumors that was in effect at the time. We did not reclassify the results of the histopathological examination in order to reveal cases of NIFTP.

### 2.3. Ultrasound Examination

We used Aloka Prosound Alpha 7 ultrasound system (ALOKA Co., Ltd., Tokyo, Japan) with a 7.5–14 MHz linear transducer. The presence of ultrasonographic malignancy risk features was evaluated prospectively, directly before selecting nodules for FNA, by experienced sonographers (with a minimum of ten years’ experience), according to a unified pattern. Notably, all the sonographers are doctors experienced not only in diagnosing but also in the treatment of thyroid diseases, which was shown to enhance the proper interpretation of sonographic images [16]. All details of the examined nodules were stored in a custom computer database, including the presence of sonographic features relevant for EU-TIRADS category determination: (a) hypoechogenicity as compared to the normal thyroid; (b) marked hypoechogenicity (i.e., more hypoechoic than the strap muscles); (c) suspicious shape (taller than wide or round); (d) irregular margins (spiculated, microlobulated or suggesting extrathyroidal extension); (e) microcalcifications (calcifications located in the solid component of a nodule, with no posterior shadowing, not larger than 1 mm); (f) spongiform composition of an entire nodule; (g) nodule showing pure cystic echostructure, composed entirely or nearly entirely of liquid. In the case of features related to echogenicity, the lowest echogenicity irrespective of its volume share was considered. Using the above-mentioned features, we assigned all examined thyroid nodules into specific categories of EU-TIRADS [14]. The system defines 5 categories: EU-TIRADS 1 denotes a US examination with no thyroid nodules found; EU-TIRADS 2 (benign category) includes pure cysts and entirely spongiform nodules; EU-TIRADS 3 (low-risk category) includes isoechoic or hyperechoic nodules that show no features of high risk of malignancy; EU-TIRADS 4 (intermediate-risk category) includes mildly hypoechoic nodules without any feature of high risk; EU-TIRADS 5 (high-risk category) includes nodules that show at least 1 of the following: suspicious shape, irregular margins, microcalcifications, or marked hypoechogenicity.

### 2.4. Analyzed Variables

The analysis was started with the evaluation of the distribution of AUS and FLUS nodules among particular EU-TIRADS categories, both for all examined nodules and for excised nodules only, considering the division of the nodules into benign lesions and cancers in the postoperative histopathological examination. That allowed us to determine RoM of AUS and FLUS nodules classified into particular EU-TIRADS category. The RoM was considered as a range, with the lower limit defined as a quotient of the number of histopathologically verified cancers and the total number of nodules classified into analyzed EU-TIRADS category (of all patients: surgically treated or not). The upper limit of RoM was defined as the incidence of cancers in nodules of surgically treated patients only. Next, the distribution of AUS and FLUS nodules among EU-TIRADS categories and mean size of nodules were compared between groups treated surgically after first FNA and those referred to rFNA, as well as between patients after rFNA—treated or untreated surgically. RoM of AUS nodules excised without rFNA or after rFNA was compared and analogical comparison was made for FLUS nodules. Then, the efficiency of EU-TIRADS in distinguishing cancers from benign nodules was assessed by analyzing ROC curves separately for AUS and FLUS nodules excised without rFNA or after rFNA. The cut-off category of EU-TIRADS with the highest accuracy (ACC) was also identified separately for AUS and FLUS excised without rFNA or after rFNA. The efficiency of the determined thresholds was evaluated with the use of the following statistical measures: the sensitivity (SEN), the specificity (SPC), ACC, the positive predictive value (PPV) and the negative predictive value (NPV). The percentage of nodules that satisfied the given criteria was also determined. The odds ratio (OR) for the established cut-off categories was assessed with the use of logistic regression analysis.

The efficiency of rFNA was also analyzed. The distribution of rFNA results among particular categories of cytological outcomes in BSRTC was evaluated for all nodules subjected to rFNA and separately for nodules that were eventually excised in respect of the division of the nodules into benign lesions and cancers as diagnosed with the postoperative histopathological examination. Using those data, we determined RoM of AUS and FLUS nodules classified into particular BSRTC categories of rFNA. The calculated risks were compared to RoM of AUS and FLUS nodules of patients treated surgically without rFNA. Comparison matrices for the distributions of EU-TIRADS categories and rFNA outcome categories were made for AUS and FLUS nodules separately. Next, analogically to the analysis of EU-TIRADS, the efficiency of rFNA in distinguishing cancers from benign nodules was assessed by analyzing ROC curves separately for AUS and FLUS nodules excised after rFNA. The cut-off category of BSRTC with the highest ACC was also identified separately for AUS and FLUS. The efficiency of the determined thresholds was presented as SEN, SPC, ACC, PPV and NPV. The percentage of nodules that satisfied the given criteria was determined and OR value was calculated for the established cut-off categories. 

Finally, we checked how the addition of EU-TIRADS category analysis to rFNA outcome analysis affected SEN, SPC, ACC, PPV and NPV of AUS and FLUS diagnostics in comparison to rFNA analysis alone. 

The statistical analysis was performed with Dell Statistica (data analysis software system), version 13, Dell Inc. (2016), Round Rock, TX, USA. The comparison of frequency distributions was performed with chi2 test (with modifications appropriate for the number of analyzed cases). The Kruskal–Wallis test was used for the comparison of continuous variables between groups. The value of 0.05 was assumed as the level of significance. 

The study protocol was approved by the local Bioethics Committee. According to the Committee’s approval neither patient’s approval nor the informed consent for our review of patients’ clinical data and FNA results were needed.

## 3. Results

### 3.1. Effectiveness of EU-TIRADS 

Table 2 shows the distribution of AUS and FLUS nodules among particular categories of EU-TIRADS. The nodules classified into category 5 of EU-TIRADS had a strongly increased risk of malignancy, and OR value was several times higher for AUS nodules than FLUS nodules, OR (95% CI): 61.9 (7.1–540.1), *p* < 0.0001 and 10.9 (5.8–20.7), *p* < 0.0001, respectively. RoM of AUS nodules classified into category 5 was significantly higher than RoM of FLUS nodules of that category: 65.2–93.8 vs. 14.8–53.1 (*p* < 0.0001 for the lower limit of RoM and *p* = 0.0085 for the upper limit of RoM). A similar regularity was observed in the case of category 4: 15.6–38.5 vs. 3.5–11.1 (*p* = 0.0006 and *p* = 0.0033, respectively) (Table 2).

The AUS nodules that were excised directly after first FNA fell into category 5 of EU-TIRADS more often than their counterparts subjected to rFNA: 29.4% vs. 12.2% (*p* = 0.0255) (Table S2). Likewise, AUS nodules excised after rFNA were classified into category 5 of EU-TIRDAS more frequently than their counterparts subjected to rFNA and then observed: 26.1% vs. 6.8% (*p* = 0.0429) (Table S3). There were no analogous differences in the case of FLUS nodules. On the other hand, FLUS nodules that were removed surgically had larger volumes that their non-excised counterparts: 7.5 cm^3^ vs. 3.7 cm^3^ (*p* < 0.0001) for nodules excised after first FNA and 5.3 cm^3^ vs. 3.4 cm^3^ (*p* = 0.0368) for nodules excised after rFNA (Table S4). There were no such differences in the volume of AUS nodules.

There was no significant difference in RoM of FLUS nodules excised after first FNA or after rFNA, but the upper limit of RoM of category 5 FLUS nodules was almost twice as high in nodules excised after rFNA than in those excised after first FNA: 75.0% vs. 42.4% (*p* = 0.0661)—see Table 3. In the case of AUS nodules, the most prominent, but still not significant, differences in RoM were observed between nodules of category 4. The nodules excised after rFNA showed RoM in the range 20.8–62.5%, while the risk of malignancy calculated for the nodules excised directly after first FNA was 0.0% (90.0% cancers corresponded to AUS nodules of category 5 EU-TIRADS, and there was no cancer among AUS nodules of category 4). 

The evaluation of EU-TIRADS showed higher diagnostic effectiveness (as measured with AUC) for AUS nodules than for FLUS nodules, especially in the case of nodules excised directly after first FNA (Table 4). In this subgroup, the ACC of EU-TIRADS was the highest when the cut-off was set at category 5, both for AUS and FLUS nodules. However, with that threshold, uniformly high SPC and NPV (>90% for both AUS and FLUS nodules) were accompanied by variable SEN and PPV, which were significantly higher for AUS nodules than for FLUS nodules, SEN: 90.0% vs. 31.1% (*p* = 0.0022) and PPV: 90.0% vs. 42.4% (*p* = 0.0226), respectively. The risk of malignancy in an AUS nodule significantly increased when the nodule had been classified into category 5 of EU-TIRADS, OR (95% CI): 207.0 (11.7–3767.5), *p* = 0.0003, while in the case of FLUS nodules that increase was less striking but still significant, OR (95% CI): 7.4 (3.4–16.3), *p* < 0.0001.

In the subgroup of nodules excised after rFNA, the highest ACC of EU-TIRADS was noted when the cut-off was set at category 5 for FLUS nodules, but category 4 for AUS nodules (see Figure S2). When the threshold was set at category 4, SEN was the same for AUS and FLUS nodules: 84.6%, but PPV was three times higher for AUS than FLUS nodules: 77.8% vs. 25.3% (*p* = 0.0003). The risk of malignancy increased significantly when the EU-TIRADS category of a nodule was over the threshold: in FLUS nodules of category 5 EU-TIRADS, OR (95% CI) was 25.9 (7.4–91.4), *p* < 0.0001, and in AUS nodules of category 4 or 5, OR was 12.8 (1.7–97.2), *p* = 0.0135.

PTC revealed in AUS nodules corresponded to category 5 of EU-TIRADS in 60.0% of cases (12 out of 20), while PTC diagnosed in FLUS nodules—in 37.1% of cases (13 out of 35) (NS). FTC was diagnosed only in FLUS nodules and 38.5% of them were classified into category 5 of EU-TIRADS (5 out of 13).

### 3.2. Effectiveness of rFNA

Outcomes of rFNA were most commonly classified again into category III BSRTC in the case of AUS nodules, and category II in the case of FLUS nodules (Table 5). Accordingly, rFNA results in the group of AUS nodules were classified into category III more often than in the case of FLUS nodules: 48.8% vs. 31.3% (*p* = 0.0012). That relation was reversed in the case of category II: 39.0% vs. 56.0% (*p* = 0.0031). There was no category IV result of rFNA in any AUS nodule. Diagnoses of category V or VI (jointly) were observed more often in outcomes of rFNA of AUS nodules than FLUS nodules, 7.3% vs. 1.3%, *p* = 0.0001. After rFNA AUS nodules were excised more frequently than FLUS nodules, AUS: 28.0% (23 out of 82) vs. FLUS: 16.2% (151 out of 934, *p* = 0.0062).

The frequency distribution of BSRTC categories of rFNA outcomes in patients treated surgically did not differ significantly between AUS and FLUS nodules. The most common category for both types of nodules was category III, AUS: 52.2%, FLUS: 49.0% (NS) (see Table 5). However, the percentage of rFNA diagnoses of category V or VI (jointly) was significantly higher in the case of AUS nodules than FLUS nodules: 21.7% vs. 7.2% (*p* < 0.0001). The mean volume of FLUS nodules that were classified into category II in rFNA and then treated surgically was significantly higher than that of their non-excised counterparts: 8.2 ± 20.7 vs. 3.4 ± 8.8 (*p* = 0.0094). There were no similar differences in the cases of other categories of rFNA outcomes, either for FLUS or AUS nodules.

RoM of all AUS nodules (excised after first FNA or rFNA) was in the range 18.1–40.4% and was higher than RoM of FLUS nodules: 4.1–13.4% (*p* < 0.0001 for both the lower and the upper limit of RoM) (Table 5). AUS nodules excised after rFNA showed a nearly twice higher rate of malignancy (the upper limit of RoM) than AUS nodules excised without rFNA: 56.5% vs. 29.4% (*p* = 0.0407). In the case of FLUS nodules, the analogous difference was smaller and insignificant: 17.2% vs. 11.9% (*p* = 0.1079). However, the detailed analysis of RoM for particular categories of rFNA showed that FLUS nodules were the type in which there were significant differences in RoM of nodules reclassified into other BSRTC categories in comparison with nodules excised without rFNA. Specifically, when rFNA of a FLUS nodule brought the diagnosis of category V or VI then the upper limit of RoM significantly increased (to 85.7 and 75.0%, respectively) in comparison to the nodules excised without rFNA (11.9%, with *p* < 0.0001 and *p* = 0.0025, respectively). There was also an increase in the lower limit of RoM (to 75.0%, *p* < 0.0001 in both cases). A significant increase in RoM was also observed when AUS was diagnosed in rFNA of a FLUS nodule: up to 15.0–42.9% (*p* < 0.0001 for the lower limit of RoM and *p* < 0.0609 for the upper limit of RoM). On the other hand, when the result of rFNA of a FLUS nodule was classified into category II then the lower limit of its RoM significantly decreased (14 times) from 5.6% to 0.4% (*p* < 0.0001). Consequently, the evaluation of rFNA category showed a higher effectiveness (as measured with AUC) in distinguishing cancers from benign lesions in the case of FLUS nodules than AUS nodules, AUC (95% CI): 0.733 (0.6–0.8), *p* < 0.0001 vs. 0.658 (0.4–0.9), *p* = 0.1719, respectively. 

The highest ACC of rFNA was reached with the cut-off set at category V for both AUS and FLUS nodules: 65.2% vs. 87.4% (*p* = 0.0062) (Figure S2). At that threshold, RoM for both types of nodules significantly increased in comparison with nodules excised without rFNA. In the case of AUS nodules, RoM increased from 22.2–29.4% to 83.3–100% (*p* < 0.05 for both limits of RoM), while in the case of FLUS nodules—from 5.6–11.9% to 75.0–81.8% (*p* < 0.0001 for both limits of RoM). The threshold set at category V was denoted with ‘rFNA-T1’. In the case of FLUS nodules, there was also a significant increase in RoM to the range of 31.0–46.4% when the cut-off was set at the level of AUS diagnosis in rFNA (*p* < 0.0001 for both limits of RoM). A similar effect was observed for AUS nodules with the cut-off set category III of any subcategory: RoM increased to the range of 21.7–58.8% (*p* = 0.0427 for the upper limit of RoM, NS for the lower limit). Those thresholds were denoted with ‘rFNA-T2’. 

Using the rFNA-T1 threshold, we found SEN to be low and similar for both types of nodules, AUS: 38.5%, FLUS: 34.6%, while SPC was high, AUS: 100.0%, FLUS: 98.4% (Table S5). There were notable differences in the NPV, which was significantly lower for AUS nodules than FLUS nodules: 55.6% vs. 87.9% (*p* = 0.0004). FLUS nodules that satisfied the rFNA-T1 threshold showed a significantly increased rate of malignancy: OR (95% CI): 32.6 (6.5–163.5, *p* < 0.0001), and in the case of AUS nodules, that effect was even more pronounced although statistically insignificant, OR > 100 (*p* = 0.9977). 

The use of rFNA-T2 threshold instead of rFNA-T1 led to an SEN twice as high for AUS nodules: 76.9%, but also a markedly lowered SPC, to 30.0%. For FLUS nodules, the increase in SEN was lower, up to 50.0%, but SPC still remained high (88.0%, *p* < 0.0001 vs. AUS). The change of the threshold did not significantly influence NPV or ACC, and their values remained lower for AUS than FLUS nodules, NPV: 50.0% vs. 89.4% (*p* = 0.0259), ACC: 56.5% vs. 81.5% (*p* = 0.0070). FLUS nodules that satisfied the rFNA-T2 threshold showed an increased risk of malignancy: OR (95% CI): 7.3 (2.9–18.8, *p* < 0.0001; in the case of AUS nodules OR was lower and the increase in the risk was insignificant (OR = 1.4, *p* = 0.7084).

Regardless of the threshold used, a higher percentage of AUS nodules than FLUS nodules reached the cut-off category: at rFNA-T1 threshold, the percentage was twice as high (21.7% vs. 7.3%, *p* = 0.0254), and at rFNA-T2 threshold—three times higher (73.9% vs. 18.5%, *p* < 0.0001). 

### 3.3. Effectiveness of rFNA and EU-TIRADS Combined

Table S6 shows the comparison matrices of the distributions of EU-TIRADS categories and categories of rFNA outcomes separately for excised AUS and FLUS nodules. In both types of nodules, when category II was diagnosed in rFNA and a nodule was classified into category 3 of EU-TIRADS, then the nodule was benign in 100% of cases. On the other hand, an AUS nodule classified into category 5 of EU-TIRADS turned out to be a cancer in 100% of cases.

The comparison of diagnostic effectiveness of rFNA and its combination with EU-TIRADS showed that the highest ACC in the case of AUS nodules could be reached when rFNA-T1 threshold was alternatively combined with EU-TIRADS category, with the cut-off value set at category 4 or 5 (Table 6). ACC was the same for both cut-off categories: 82.6%, but SEN and SPC differed. The highest and significant increase in SEN was observed (in comparison with the isolated rFNA evaluation with category V as the cut-off value) when the rFNA-T1 threshold was alternatively combined with EU-TIRADS 4. Then, SEN increased to 92.3% (*p* = 0.0134 vs. rFNA(1): 38.5%), with SPC amounting to 70%. A similar SEN could be achieved with the combination of rFNA evaluation using the rFNA-T2 threshold with EU-TIRADS evaluation with the cut-off set at category 5 or 4 (SEN: 92.3% and 100.0%, respectively), but such a combination led to a very low SPC (30.0% and 20.0%, respectively) and a very high number of nodules fulfilling the criteria for malignancy (82.6% and 91.3%, respectively). The combined criterion (rFNA-T1 or EU-TIRADS 4) did not increase the number of positive nodules in comparison with rFNA-T2 criterion (65.2% vs. 73.9%). Furthermore, such nodules had a significantly increased risk of malignancy, OR (95% CI): 28.0 (2.4–323.7), *p* = 0.0076.

In the case of FLUS nodules, the highest ACC—88.7%—was achieved with the alternative combination of rFNA-T1 threshold with EU-TIRADS category 5 threshold. Such a combination resulted in an SEN of 57.7% and an SPC of 95.2%. Only 13.9% of nodules fulfilled that criterion and they had a significantly increased risk of malignancy, OR (95% CI): 27.0 (8.7–83.8), *p* < 0.0001. Similar ACCs (88.1%) and SPCs (96.8%) were achieved with the criterion based on EU-TIRADS only with the cut-off at category 5, but SEN was lower by 11.5 percentage points in comparison with the combined criterion. A significant increase in SEN was assured with criteria based on EU-TIRADS evaluation with the threshold set at category 4 in combination with any of two established thresholds for rFNA. However, regardless of the threshold for rFNA, setting the EU-TIRADS cut-off value at category 4 led to a decrease of SPC under 50% and a decrease of ACC to the level of 55% for rFNA-T1 or 51.7% for rFNA-T2.

## 4. Discussion

It is still a serious challenge to produce a commonly accepted recommendation on the management of patients with thyroid nodules classified into category III. The introduction of molecular tests run on the material obtained during FNA brought some hope in that respect. However, the published studies on the performance of such tests did not show results satisfactory enough to justify the high costs of multigene panels. Attempts to use novel ultrasound techniques, such as 3D-ultrasound, shear wave elastography or contrast-enhanced ultrasonography, while promising, did not bring any breakthrough in the area [17,18]. Consequently, in the majority of endocrine centers, clinical decisions regarding such patients are based on repeat FNA and ultrasound imaging, which serve to narrow the estimated range of RoM of the nodule.

Nodules of category III do not constitute a uniform group and our results clearly indicate that it is necessary to distinguish nodules that present features of cytologic atypia (more commonly corresponding to PTC) from nodules without such features, usually presenting architectural abnormalities typical of follicular lesions. Both types of nodules mentioned differ significantly in their associated RoM. Moreover, rFNA and SRSs show different effectiveness in the assessment of such nodules. The ultrasound diagnostics of nodules presenting architectural atypia is more challenging. At the same time, rFNA of these nodules brings a diagnosis of a benign lesion more often than in the case of nodules with cytologic/nuclear atypia, and such a result of rFNA markedly lowers the RoM of the nodule. Nodules with cytologic/nuclear atypia display quite opposite qualities: the diagnosis of a benign lesion in rFNA is not only less common but it does not lower RoM of the nodule. Moreover, categories of rFNA outcome that strongly suggest the necessity of surgical treatment (i.e., V and VI BSRTC) are observed more frequently among nodules with cytologic/nuclear atypia than in the case of nodules with architectural atypia. Similarly, the ultrasonographic presentation typical of malignancy is also more common among malignant nodules with cytologic/nuclear atypia than those with architectural atypia. Unfortunately, rFNA of AUS nodules brings a result of category III again in about half the cases, and when such a result of rFNA is considered as an indication for surgery, it inevitably leads to the unnecessary excision of many benign nodules. 

The diagnostic effectiveness may be improved when indications for surgery are based on the joint criteria, using both the results of rFNA and the assessment of sonographic risk with SRSs, but it is important to select optimal diagnostic goals. In the case of nodules with cytologic/nuclear atypia and a high RoM, the main goal should be optimization of SEN of the following diagnostic procedures. Contrarily, in the case of nodules with architectural atypia, which are associated with a markedly lower RoM, the optimization of SPC and NPV should be of primary concern, especially in populations in which such nodules predominate.

Observing these assumptions, we showed that the best relation between SEN and SPC in the case of AUS nodules could be achieved if a nodule was regarded positive when rFNA result was at least of category V BSRTC or the nodule was classified into category 4 (or 5) of EU-TIRADS. Such joint criteria assured higher SEN (92.3%) than the criterion based solely on repeat diagnosis of category III in FNA, with significantly higher ACC (82.6%) and lower percentage of positive nodules (65.2%). In the case of FLUS nodules, the optimal criterion was found to be category V in rFNA or category 5 of EU-TIRADS. Such a combination led to optimal SPC (95.2%), NPV (91.5%) and assured higher SEN (57.7%) than the criterion based solely on a significant increase in RoM indicated by rFNA, i.e., with the threshold set at the AUS diagnosis in rFNA. Even more prominent differences were observed when FLUS nodules were regarded positive if the rFNA result was of category III once again, without taking into account the type of atypia. Such an approach is taken in many recommendations. In our material, it would have resulted in a high rate of benign nodules being excised (57.6% instead of 4.8%). That rate is also affected by a low RoM of FLUS nodules classified into category IV in rFNA in our material. It is a phenomenon typical of areas that suffer from iodine deficiency, where there is a high number of benign follicular nodules. It should be underlined that both joined criteria we identified are more effective than criteria based on categories V and VI rFNA only, which show low SEN. Following the obtained results, we made a diagram that illustrates the proposed optimal diagnostic path for thyroid nodules with cytologic atypia or architectural atypia (Figure S3).

It may be surprising that the joint criterion for AUS nodules uses category 4 EU-TIRADS but not 5. One could argue that AUS nodules correspond to PTC more often than FLUS nodules and their presentation in ultrasonography usually classifies them into category 5 EU-TIRADS and consequently that should be the category which would assure a satisfying SEN with high SPC. However, it occurred that patients with AUS nodules that were classified into category 5 EU-TIRADS were quite often referred to surgical treatment without rFNA. Due to that preselection, AUS nodules subjected to rFNA showed ultrasound malignancy features half as often. Similar observations were made by other researchers who studied populations with the predominance of nodules with cytologic atypia [19,20]. 

There are reports on the optimal strategy of selecting patients with nodules of category III in rFNA which should be treated surgically but in the majority of those studies, nodules with different types of atypia were evaluated jointly. Their authors usually did not report the proportion of each type of nodules (AUS vs. FLUS) in their material. Consequently, there are marked contradictions between their results. Moreover, some studies were focused on the comparison of malignancy rates between nodules in patients treated surgically directly after first FNA and in those treated only after rFNA. Such a comparison seems to reveal a kind of misunderstanding of the diagnostic challenges that we face. It should be remembered that rFNA may lead both to an increase or to a decrease in the estimated RoM of a nodule. Patients actually referred to surgery are not necessarily those in whom rFNA had increased RoM. This is well illustrated by our group of patients with FLUS nodules. In the case of these nodules, it was the size of a nodule and not its worrying ultrasonographic presentation that usually prompted surgical treatment, not only directly after first FNA but also when rFNA brought a category II result. We can speculate that the decision to perform rFNA in such cases was motivated by an intention to identify the optimal extension of surgery which should be larger in the case of a malignancy diagnosed with rFNA. Consequently, in the case of FLUS nodules, an increased malignancy rate found in the histopathological examination of nodules with rFNA diagnosis of AUS or of category V–VI was probably counterbalanced by a decreased rate of malignancy in nodules of category II in rFNA. It is one of the probable causes of discrepancies between reported results in that respect. Some studies showed a similar frequency of cancers in patients treated surgically after or without rFNA [21,22,23]; others indicated an increased rate of malignancy after rFNA, non-significantly [8,24,25,26,27,28,29] or significantly [20,30,31,32,33] and there are reports that suggested slightly lower malignancy rates in patients undergoing surgery after rFNA in comparison to those treated directly after the diagnosis of category III [34,35].

Another source of the differences in the reported usefulness of rFNA comes from the variable effectiveness of the selection of patients that need surgery based on the first FNA. In populations with a high percentage of PTC among cancers, its characteristic ultrasonographic features help to achieve a rate of malignancy as high as over 75% in patients referred to surgery after first FNA [19,20]. That rate is even higher in patients with a repeat diagnosis of category III, in whom nodules presenting suspected ultrasound features are less common [19]. On the other hand, our observations suggest that the effectiveness of ultrasound diagnostics may be neglected due to the patient’s preference to quickly and unequivocally resolve diagnostic uncertainty with surgery, especially in the case of nodules with cytologic/nuclear atypia that have higher RoM. In our material, as much as 56% of AUS nodules excised without rFNA corresponded to the low risk category in EU-TIRADS classification. Only 5.3% of them were eventually diagnosed as cancers in comparison to the 90% rate of cancers among nodules of category 5 EU-TIRADS. Among FLUS nodules excised without rFNA, the percentage of the low risk category in EU-TIRADS was lower (38%), while the rate of malignancy was similar (7.7%).

That problem is closely related to the question of conditions in which surgical treatment may be safely abandoned in patients with category III nodules. We found that no cancer was histopathologically diagnosed among nodules of low risk category in EU-TIRADS that were benign lesions in rFNA. It seems that these are satisfactory premises to safely put surgical treatment aside in the case of FLUS nodules. There are still some doubts regarding AUS nodules as we found that a category II result of rFNA did not significantly lower their RoM. It should be a matter of further studies as the number of AUS nodules subjected to rFNA was relatively small in our material. With a few exceptions, other studies generally did not analyze both types of category III nodules separately. Two such studies showed that category II BSRTC in rFNA occurs more often in the case of nodules with architectural atypia than nodules with cytologic/nuclear atypia [7,9], which is in agreement with our observations. Another study did not indicate any difference in the rates of particular categories of rFNA outcomes between nodules with or without cytologic/nuclear atypia [5]. Many papers that described nodules of category III without their subcategorization showed a marked variation in the rate of rFNA category II results between diagnostic centers, from 36 to 70% [9,19,20,21,22,23,24,25,27,30,31,32,34,36,37]. These reports also differed in the observed consequences of the category II diagnosis in rFNA. Brandler et al. [25] showed a 100% safety of putting surgical treatment aside but only four of the evaluated nodules had been verified histopathologically. Others indicated that there was a statistically significant decrease in the rate of non-neoplastic lesions [22] and benign lesions [8,27,38] in the surgical pathology material. There are also reports suggesting that the malignancy risk in the case of a benign aspirate after the initial AUS/FLUS diagnosis remain high—18%, according to Sullivan et al. [39] or 29%, according to VanderLaan et al. [24]—and it does not differ significantly from that observed in patients treated surgically without rFNA. Renshaw found that the malignancy rate of such nodules falls in the range between the rate for nodules with a single diagnosis of category II (1.7%) and a single diagnosis of category III (24.5%) [40]. 

Obviously, the differences in the reported effectiveness of rFNA, both in revealing malignancy or in confirming benignity of a nodule, are also a consequence of low reproducibility of equivocal cytological diagnoses. The latter comes from some imprecisions in the definitions of these categories as well as from the diagnostic center-specific susceptibility to the degree of atypia [41]. 

When reviewing studies on the usefulness of SRSs, we face the same problem as in the case of reports on the effectiveness of rFNA, i.e., the lack of distinction between nodules with and without cytologic/nuclear atypia. In one of the few exceptions, Lee et al. found the American Thyroid Association (ATA) guidelines to be useful only in the AUS subcategory of category III [42]. Analogous conclusions were drawn by Yoon JH et al. [43] and Baser et al. [44] in relation to the system suggested by Kwak and Kim et al. [45] using the Korean Thyroid Imaging Reporting and Data System. Plainly, these studies showed similar results to our present analysis. The latter report showed that FTCs more often than PTCs were classified into categories other than the high risk one. For the same reason, we observed higher than expected (2.1–7.2% vs. 2–4%) values of RoM for FLUS nodules of the low risk category in EU-TIRADS, the group in which all identified FTC were located. We did not find any similar studies concerning EU-TIRADS, despite the fact that both our studies as well as the study by Castellana et al. (2020) indicated that EU-TIRADS was characterized by a low frequency of missed FTCs in comparison to other systems [11,46]. Furthermore, it is FTC that is more common in the case of nodules with architectural atypia. 

Some studies did not focus on any particular SRS, but only examined the features considered in those systems as key features for the identification of high risk nodules. In one of these such studies, Rosario [9] showed that a suspicious ultrasonographic presentation was found twice as often in AUS nodules as in FLUS nodules. Topaloglu et al. [47] found that malignant and benign AUS nodules differed in the frequency of suspicious shape or microcalcification, while benign and malignant FLUS nodules showed similar rates of all parameters (including irregular margins and hypoechogenicity). Çuhaci et al. [48] indicated that the only predictive features of malignancy were hypoechogenicity in the AUS group and peripheral vascularization in the FLUS group. 

Other researchers evaluated all category III nodules jointly, without any consideration for the type of atypia. The majority of their reports that showed the usefulness of various SRSs in detecting malignancy came from the studies performed on populations with high iodine consumption and consequently, a high percentage of PTC among cancers. Tang et al. (2017) found that the sonographic system recommended by ATA was useful to predict malignancy in FLUS/AUS nodules [49]. Similar observations on that system came from Valderrabano et al. (2018) [50], Hoong H.S. et al. [51], Ahmadi et al. (2019) [52]. The latter study included not only ATA guidelines but also ACR-TIRADS, the system recommended by the American College of Radiology (ACR) and its authors concluded that both systems can be used equally successfully to risk-stratify nodules with indeterminate cytology, including category III nodules. Similar results were also reported by Kamaya et al. [53] and Ulisse et al. [54] in relation to the system proposed by Kwak as well as by Hong M.J et al. [55], Hong H.S. et al. [51], Suh et al. [56] and Jeong et al. [57] in relation to the system created by the Korean Society of Thyroid Radiology. Contrary observations were made by Grani et al. [58] who examined the usefulness of the systems recommended by ATA and by Korean radiologists in an Italian population. They found that neither of those systems was effective in diagnosing malignancy but they were helpful in the high-confidence exclusion of malignancy in nodules with indeterminate cytology, according to the 2010 Italian Consensus on Thyroid Cytology Criteria. Similarly, Barbosa et al. [59] found in a Brazilian population that ACR-TIRADS and ATA guidelines may help guide the management of indeterminate thyroid nodules, suggesting a conservative approach to nodules with a low-risk ultrasound suspicion. On the other hand, Chaigneau et al. (2015) found in a French population that the risk stratification with French TIRADS (which resembles the current EU-TIRADS) was not significant in Bethesda III nodules [60]. The authors believed, as we do in the case of FLUS nodules, that the reason was a relatively low percentage of PTC, especially its classical variant. FVPTC and FTC have been reported to often show no strong ultrasonographic risk features, contrary to the classical variant of PTC [46,60,61]. In our study, we did not have information on the specific subtype of PTC, nor did we repeat the examination of histopathological slides to possibly reclassify some diagnoses into NIFTP. However, we observed that PTCs corresponding to FLUS nodules were nearly half as often classified into the high risk category of EU-TIRADS than PTCs corresponding to AUS nodules—which may be a result of different proportions of FVPTC in both groups. Maia et al. analyzed a system resembling the French TIRADS system (with the addition of vascularity criteria) and declared its usefulness in the group of nodules of category III–V for the stratification of malignancy risk [62]. However, a more detailed analysis of their data shows that the conclusion is not supported by obtained results; in the case of category III nodules, the risk of malignancy for particular categories of the analyzed system (3, 4A and 4B) was very close and in the range of 8.7–10%, and there was only one nodule classified into category 5 (of the highest risk), which eventually was found to be benign. Sahli et al. [63] found that ACR-TIRADS was a poor predictor of final surgical pathology in the group of cytologically indeterminate (category III and IV of BSRTC) and Afirma-suspicious nodules. Similarly, Trimboli et al. [12] found that SRSs had suboptimal diagnostic accuracy in the diagnostics of indetermined nodules, including category III nodules. In our opinion, further analyses of the usefulness of SRSs in the diagnostics of nodules with indetermined cytology are necessary, but they need to handle nodules of category III separately, and furthermore, consider groups of nodules with and without cytologic/nuclear atypia as they constitute different entities.

There are some limitations in our study. We did not review histopathological specimens and did not know the actual incidence of NIFTP or other borderline tumors in our material. Reliable data on the presence of clinical features indicative of an increased risk of thyroid cancer were not available in every case, therefore, we did not analyze such features. Additionally, the group of patients included in the study showed some characteristic qualities of a population that had been exposed to iodine deficiency—a high percentage of nodules with architectural atypia and relatively frequent surgical treatment due to the size of goiter but not alarming results of rFNA or ultrasound imaging. An important advantage of our study consists in determining the definite diagnosis with postoperative histopathological examination, which is especially important as differentiated thyroid cancers are known for long periods without any progression that could be identified in the clinical follow up. On the other hand, such a solid determination of the diagnosis overestimates the actual rate of malignancy—a number of benign nodules never reach confirmed diagnosis. Thus, we decided to determine RoM ranges. Another advantage of our study was performing the evaluation of US malignancy features and of EU-TIRADS category prior to biopsy. Therefore, the results of FNA and histopathological examination did not influence that evaluation.

## 5. Conclusions

Nodules of category III BSRTC with cytologic/nuclear atypia show different RoM than nodules without that type of atypia. SRSs and rFNA have different effectiveness in both types of nodules. The precision of distinguishing cancers from benign nodules is better for SRSs than rFNA in the case of nodules with cytologic/nuclear atypia, while that relationship is opposite in the case of nodules with architectural atypia. It is safe to put surgical treatment of an FLUS nodule aside when the rFNA outcome shows a benign lesion (category II), especially with the low risk category of EU-TIRADS. It is not the case with AUS nodules as a category II outcome of rFNA has not been proved to lower their RoM. The high risk category of SRSs in the case of AUS nodules is a strong indication for surgical treatment right after first FNA. Indeed, these nodules are frequently being excised at this stage. That is why we should adopt SRS threshold at category 4: intermediate risk EU-TIRADS when re-evaluating indications for surgery after rFNA of an AUS nodule. The joint criteria of EU-TIRADS category 4 (or higher) or rFNA category V (or higher) offer better separation of benign and malignant AUS nodules than any criterion based solely on rFNA category. Similarly, in the case of FLUS nodules, best effectiveness (i.e., optimal NPV and SPC) may be achieved with the use of the combined criteria: category V BSRTC in rFNA or category 5 (high risk) EU-TIRADS.

## Figures and Tables

**Table 1:** Demographic data of the patients with nodules with cytologic/nuclear atypia (AUS) and nodules with architectural atypia (FLUS), including data on nodules subjected to repeat FNA (rFNA) or surgical treatment.

Variable	AUS	FLUS	*p*
No. of all patients	125	1723	
Age, mean ± SD (years)	60.2 ± 14.1	58.8 ± 13.8	0.2697
No./% of males	21/16.8	196/11.4	0.0689
Number of all nodules	127	1739	
Volume of nodules, mean ± SD (cm^3^)	5.3 ± 15.5	5.1 ± 12.9	0.8379
No./% of nodules examined with rFNA	82/64.6	934/53.7	0.0177
No./% of nodules excised after rFNA	23/28.0	151/16.2	0.0062
No./% of excised nodules	57/44.9	528/30.4	0.0007
No./% of excised nodules without rFNA	34/59.6	377/71.4	0.0652
No./% of excised nodules after rFNA	23/40.4	151/28.2	
No./% of cancers	23	71	
No./% of PTC among cancers	20/87.0	35/49.3	0.0033
Age of patients with cancers, mean ± SD (years)	53.6 ± 16.8	53.5 ± 15.1	0.9692
Age of patients with benign nodules, mean ± SD (years)	58.9 ± 11.6 #	54.1 ± 12.7 ##	0.0318
No./% of males among patients with cancers	5/21.7	12/17.4	0.6417
No./% of males among patients with benign nodules	3/8.8 #	55/12.3 ##	0.5452

#—NS vs. patients with cancer in AUS nodules, ##—NS vs. patients with cancers in FLUS nodules.

**Table 2:** Distribution of AUS and FLUS nodules among particular categories of EU-TIRADS and their associated risk of malignancy (RoM).

Category of EU-TIRADS	AUS Nodules				FLUS Nodules			
	All 127	Excised 57	Cancers 23	RoM	All 1739	Excised 528	Cancers 71	RoM
EU-TIRADS 2	0/0.0	0/0.0	0/0.0	0.0	3/0.2	1/0.2	0/0.0	0.0
EU-TIRADS 3	72/56.7 a	28/49.1	3/13.0	4.2–10.7	714/41.1	207/39.2	15/21.1	2.1–7.2
EU-TIRADS 4	32/25.2 b	13/22.8 b	5/21.7 c	15.6–38.5	846/48.6	271/51.3	30/42.2	3.5–11.1
EU-TIRADS 5	23/18.1 c	16/28.1 b	15/65.2 d	65.2–93.8	176/10.1	49/9.3	26/36.6	14.8–53.1

a—*p* < 0.001 vs. FLUS; b—*p* < 0.0001 vs. FLUS; c—*p* < 0.005 vs. FLUS; d—*p* < 0.01 vs. FLUS.

**Table 3:** Comparison of the risk of malignancy (RoM) in AUS and FLUS nodules in patients treated surgically without or after rFNA for particular EU-TIRADS categories.

**Category of** **EU-TIRADS**	**AUS Nodules**				**FLUS Nodules**			
	**without rFNA**							
	**All** **45**	**Excised** **34**	**Cancers** **10**	**RoM**	**All** **805**	**Excised** **377**	**Cancers** **45**	**RoM**
EU-TIRADS 2	0/0.0	0/0.0	0/0.0	-	3/0.4	0/0.0	0/0.0	0.0
EU-TIRADS 3	24/53.3	19/55.9	1/10.0	4.2–5.3	324/40.3	143/37.9	11/24.4	3.4–7.7
EU-TIRADS 4	8/17.8	5/14.7	0/0.0 a	0.0	402/49.9	200/53.1	20/44.4	5.0–10.0
EU-TIRADS 5	13/28.9	10/29.4	9/90.0 b	69.2–90.0	76/9.4	33/8.8	14/31.1	18.4–42.4
	**after rFNA**							
	**All** **82**	**Excised** **23**	**Cancers** **13**	**RoM**	**All** **934**	**Excised** **151**	**Cancers** **26**	**RoM**
EU-TIRADS 2	0/0.0	0/0.0	0/0.0	-	0/0.0	0/0.0	0/0.0	-
EU-TIRADS 3	48/58.5	9/39.1	2/15.4	4.2–22.2	390/41.8	64/42.4	4/15.4	1.0–6.3
EU-TIRADS 4	24/29.3	8/34.8	5/38.5	20.8–62.5	443/47.4	71/47.0	10/38.5	2.2–14.1
EU-TIRADS 5	10/12.2	6/26.1	6/46.2	60.0–100.0	101/10.8	16/10.6	12/46.2	11.9–75.0

a—*p* < 0.05 vs. cancers in FLUS nodules operated without rFNA; b—*p* < 0.001 vs. cancers in FLUS nodules operated without rFNA.

**Table 4:** Diagnostic effectiveness of EU-TIRADS for all excised AUS and FLUS nodules, evaluated for nodules excised without and after rFNA. Cut-off categories showing the highest ACC are marked with bold.

	**AUS Nodules**					
	**All Excised**		**Excised without rFNA**		**Excised after rFNA**	
	EU-TIRADS 5	EU-TIRADS 4	**EU-TIRADS** **5**	EU-TIRADS 4	EU-TIRADS 5	**EU-TIRADS** **4**
SEN	65.2	87.0	90.0	90.0	46.2	84.6
SPC	97.1	73.5	95.8	75.0	100.0	70.0
ACC	84.2	78.9	94.1	79.4	69.6	78.3
PPV	93.8	69.0	90.0	60.0	100.0	78.6
NPV	80.5	89.3	95.8	94.7	58.8	77.8
No./% of nodules	16/28.1	29/50.9	10/29.4	15/44.1	6/26.1	14/60.9
AUC (CI 95%) *p*	0.876 (0.774–0.977) <0.0001		0.919 (0.788–1.0) <0.0001		0.842 (0.679–1.0) <0.0001	
	**FLUS Nodules**					
	**All Excised**		**Excised without rFNA**		**Excised after rFNA**	
	EU-TIRADS 5	EU-TIRADS 4	**EU-TIRADS** **5**	EU-TIRADS 4	**EU-TIRADS** **5**	EU-TIRADS 4
SEN	36.6	78.9	31.1	75.6	46.2	84.6
SPC	95.0	42.2	94.3	39.9	96.8	48.0
ACC	87.1	47.2	86.7	44.1	88.1	54.3
PPV	53.1	17.5	42.4	14.6	75.0	25.3
NPV	90.6	92.8	91.0	92.4	89.6	93.8
No./% of nodules	49/9.3	320/60.6	33/8.8	233/61.8	16/10.6	87/57.6
AUC (CI 95%) *p*	0.692 (0.619–0.765) <0.0001		0.650 (0.556–0.744) 0.0017		0.770 (0.658–0.881) <0.0001	

**Table 5:** Risk of malignancy (RoM) in AUS and FLUS nodules excised without or after rFNA, including its relation to the category of rFNA outcome.

Category III Nodules		AUS Nodules				FLUS Nodules			
		All	Excised	Cancers	RoM	All	Excised	Cancers	RoM
all		127	57	23	18.1–40.4	1739	528	71	4.1–13.4
without rFNA		45/35.4	34/59.6	10/43.5	22.2–29.4	805/46.3	377/71.4	45/63.4	5.6–11.9
after rFNA		82/64.6	23/40.4	13/56.5	15.9–56.5 c	934/53.7	151/28.2	26/36.6	2.8–17.2 d
category of rFNA	I	4/4.9	1/4.3	1/7.7	25.0–100.0	97/10.4	13/8.6	1/3.8	1.0–7.7
	II	32/39.0 a	5/21.7	2/15.4	6.3–40.0	523/56.0	43/28.5	2/7.7	0.4–4.7 e
	III	40/48.8 a	12/52.2	5/38.5	12.5–41.7	292/31.3	74/49.0	13/50.0	4.4–17.6
	FLUS	11	4	2	18.2–50.0	272	67	10	3.7–14.9
	AUS	29	8	3	10.3–37.5	20	7	3	15.0–42.9 e
	IV	0/0.0	0/0.0	-	-	10/1.1	10/6.6	1/3.8	10.0–10.0
	V	4/4.9 b	3/13.0	3/23.1	75.0–100.0	8/0.9	7/4.6	6/23.1	75.0–85.7 ef
	VI	2/2.4	2/8.7	2/15.4	100.0–100.0	4/0.4	4/2.6	3/11.5	75.0–75.0 eg

a—*p* < 0.005 vs. FLUS; b—*p* < 0.01 vs. FLUS; c—*p* < 0.05 vs. upper limit of RoM for AUS nodules operated without rFNA; d—*p* < 0.005 vs. lower limit of RoM for FLUS nodules operated without rFNA; e—*p* < 0.0001 vs. lower limit of RoM for FLUS nodules operated without rFNA; f—*p* < 0.0001 vs. upper limit of RoM for FLUS nodules operated without rFNA; g—*p* < 0.005 vs. upper limit of RoM for FLUS nodules operated without rFNA.

**Table 6:** Comparison of diagnostic effectiveness of rFNA and EU-TIRADS with their combination for particular cut-off values: rFNA-T1—the cut-off value set at category V of rFNA outcome for both AUS and FLUS nodules, rFNA-T2—the cut-off value set at category III of rFNA outcome for AUS nodules or at subcategory AUS of category III for FLUS nodules, EU-TIRADS 5—the cut-off value set at category 5 EU-TIRADS, EU-TIRADS 4—the cut-off value set at category 4 EU-TIRADS.

Measure	rFNA-T1	rFNA-T2	EU-TIRADS 5	EU-TIRADS 4	rFNA-T1 or EU-TIRADS 5	rFNA-T1 or EU-TIRADS 4	rFNA-T2 or EU-TIRADS 5	rFNA-T2 or EU-TIRADS 4	*rFNA-T1* *or* *EU-TIRADS 5* *vs. rFNA-T1* *p*	*rFNA-T1* *or* *EU-TIRADS 4* *vs. rFNA-T1* *p*	*rFNA-T2* *or* *EU-TIRADS 5* *vs. rFNA-T2* *p*	*rFNA-T2* *or* *EU-TIRADS 4* *vs. rFNA-T2* *p*
	AUS nodules											
SEN	38.5	76.9	46.2	84.6	69.2	92.3	92.3	100.0	*0.2379*	*0.0134*	*0.5867*	*0.2196*
SPC	100.0	30.0	100.0	70.0	100.0	70.0	30.0	20.0	*-*	*0.2104*	*1.0*	*0.6056*
ACC	65.2	56.5	69.6	78.3	82.6	82.6	65.2	65.2	*0.8135*	*0.8135*	*0.5457*	*0.5457*
PPV	100.0	58.8	100.0	78.6	100.0	80.0	63.2	61.9	*-*	*0.7177*	*0.7900*	*0.8468*
NPV	55.6	50.0	58.8	77.8	71.4	87.5	75.0	100.0	*0.5809*	*0.2570*	*0.8952*	*0.6733*
No./% of nodules	5/21.7	17/73.9	6/26.1	14/60.9	9/39.1	15/65.2	19/82.6	21/91.3	*0.1999*	*0.0029*	*0.7207*	*0.2432*
	FLUS nodules											
SEN	34.6	50.0	46.2	84.6	57.7	88.5	61.5	88.5	*0.0951*	*0.0002*	*0.4022*	*0.0069*
SPC	98.4	88.0	96.8	48.0	95.2	48.0	85.6	44.0	*0.2810*	*<0.0001*	*0.5751*	*<0.0001*
ACC	87.4	81.5	88.1	54.3	88.7	55.0	81.5	51.7	*0.7225*	*<0.0001*	*1.0*	*<0.0001*
PPV	81.8	46.4	75.0	25.3	71.4	26.1	47.1	24.7	*0.8299*	*0.0007*	*0.9605*	*0.0277*
NPV	87.9	89.4	89.6	93.8	91.5	95.2	91.5	94.8	*0.3215*	*0.1682*	*0.5948*	*0.3613*
No./% of nodules	11/7.3	28/18.5	16/10.6	87/57.6	21/13.9	88/58.3	34/22.5	93/61.6	*0.0615*	*<0.0001*	*0.3927*	*<0.0001*

SEN—sensitivity, SPC—specificity, ACC—accuracy, PPV—positive predictive value, NPV—negative predictive value.

## Data Availability

The data presented in this study are available on request from the corresponding authors. The data are not publicly available due to patient privacy restrictions.

## Supplementary Materials

The following supporting information can be downloaded at: https://www.mdpi.com/article/10.3390/cancers14184489/s1, Figure S1: Distribution of surgically treated nodules regarding their initial diagnosis (FLUS vs. AUS); Figure S2: ROC curve analysis of the evaluation of the diagnostic value of EU-TIRADS and rFNA categories in AUS and FLUS nodules; Figure S3: Suggested diagnostic algorithms for both types of nodules of category III (as diagnosed in first FNA); Table S1: Comparison of the incidence of particular types of cancers in AUS and FLUS nodules (as defined with first FNA); Table S2: Distribution of AUS and FLUS nodules among particular categories of EU-TIRADS in relation to the chosen path: surgery without rFNA vs. performing rFNA; Table S3: Distribution of AUS and FLUS nodules subjected to rFNA among particular categories of EU-TIRADS in relation to the chosen path: surgery vs. clinical follow-up without surgery; Table S4: Mean volumes (cm^3^ ± SD) of AUS and FLUS nodules in relation to the status of rFNA and surgery; Table S5: Comparison of diagnostic effectiveness of rFNA and EU-TIRADS in diagnostics of AUS and FLUS nodules for particular cut-off values; Table S6: Relation between category of EU-TIRADS and the outcome of rFNA for excised AUS and FLUS nodules.
