# Supplementary material for: Optimization of the Management of Category III Thyroid Nodules Using Repeat FNA and TIRADS
Source: Cancers (Basel). 2022 Sep 16;14(18):4489. doi: 10.3390/cancers14184489 (PMC9496843; doi:10.3390/cancers14184489)
Supplement: Supplementary file 1 [file cancers-14-04489-s001.zip › cancers-1899551-supplementary.pdf]

**Table S1.** Comparison of the incidence of particular types of cancers in AUS and FLUS nodules (as defined with first FNA).

| Type of malignancy | AUS 23 | FLUS 71 |
|---|---|---|
| | [number/%] | |
| PTC | 20/87.0 | 35/49.3 |
| NIFTP | - | 2/2.8 |
| FTC | - | 13/18.3 |
| FT-UMP | - | 5/7.0 |
| HTC | - | 7/9.9 |
| PDTC | - | 1/1.4- |
| ATC | 1/4.3 | 2/2.8 |
| MTC | 1/4.3 | 3/4.2 |
| ML | 1/4.3 | |
| ANG | | 1/1.4 |
| PC | - | 1/1.4 |
| ST | - | 1/1.4 |

PTC—papillary thyroid carcinoma, NIFTP - non-invasive follicular thyroid neoplasm with papillary like nuclear features, FTC follicular thyroid carcinoma, FT-UMP—follicular tumor of uncertain malignant potential, HTC—Hurthle cell thyroid carcinoma, PDTC—poorly differentiated thyroid carcinoma, ATC—anaplastic thyroid carcinoma, MTC—medullary thyroid carcinoma, ML – malignant lymphoma, ANG—angiosarcoma, PC—parathyroid carcinoma, ST—secondary tumor

**Table S2.** Distribution of AUS and FLUS nodules among particular categories of EU-TIRADS in relation to the chosen path: surgery without rFNA vs performing rFNA

| | AUS nodules [No./%] | | | FLUS nodules [No./%] | | |
|---|---|---|---|---|---|---|
| | excised without rFNA | examined with rFNA | *p* | excised without rFNA | examined with rFNA | *p* |
| EU-TIRADS 2 | 0/0.0 | 0/0.0 | - | 0/0.0 | 0/0.0 | - |
| EU-TIRADS 3 | 19/55.9 | 48/58.5 | *0.7922* | 143/37.9 | 390/41.8 | *0.2019* |
| EU-TIRADS 4 | 5/14.7 | 24/29.3 | *0.0992* | 200/53.1 | 443/47.4 | *0.0654* |
| EU-TIRADS 5 | 10/29.4 | 10/12.2 | ***0.0255*** | 33/8.8 | 101/10.8 | *0.2650* |
| All | 34 | 82 | | 377 | 934 | |

**Table S3.** Distribution of AUS and FLUS nodules subjected to rFNA among particular categories of EU-TIRADS in relation to the chosen path: surgery vs clinical follow-up without surgery

| | **AUS nodules examined with rFNA [No./%]** | | | **FLUS nodules examined with rFNA [No./%]** | | |
|---|---|---|---|---|---|---|
| | **excised** | **non excised** | ***p*** | **excised** | **non excised** | ***p*** |
| EU-TIRADS 2 | 0/0.0 | 0/0.0 | - | 0/0.0 | 0/0.0 | |
| EU-TIRADS 3 | 9/39.1 | 39/66.1 | ***0.0259*** | 64/42.4 | 326/41.6 | *0.8643* |
| EU-TIRADS 4 | 8/34.8 | 16/27.1 | *0.4932* | 71/47.0 | 372/47.5 | *0.9121* |
| EU-TIRADS 5 | 6/26.1 | 4/6.8 | ***0.0429*** | 16/10.6 | 85/10.9 | *0.9251* |
| All | 23 | 59 | | 151 | 783 | |

**Table S4.** Mean volumes [$cm^3 \pm SD$] of AUS and FLUS nodules in relation to the status of rFNA and surgery.

| **Variable** | **AUS** | **FLUS** | ***p*** |
|---|---|---|---|
| Volume of nodules examined with rFNA | 2.9 ± 5.2 | 3.7 ± 10.5 | *0.5177* |
| Volume of nodules examined with rFNA and excised | 2.6 ± 4.1 | 5.3 ± 12.9 | *0.3319* |
| Volume of nodules examined with rFNA and not excised | 3.0 ± 5.6 | 3.4 ± 10.5 | *0.7950* |
| Volume of nodules not examined with rFNA or lost | 9.8 ± 24.6 | 6.7 ± 15.1 | *0.2109* |
| Volume of excised nodules | 5.2 ± 19.5 | 6.9 ± 14.6 | *0.4353* |
| Volume of excised nodules without rFNA | 7.0 ± 25.1 | 7.5 ± 15.2 | *0.8540* |
| Volume of excised nodules after rFNA | 2.6 ± 4.1 | 5.3 ± 12.9 | *0.3320* |
| Volume of not excised nodules or lost | 5.4 ± 11.3 | 4.3 ± 9.9 | *0.4507* |

**Table S5.** Comparison of diagnostic effectiveness of rFNA and EU-TIRADS in diagnostics of AUS and FLUS nodules for particular cut-off values:
rFNA-T1 – the cut-off value set at category V of rFNA outcome for both AUS and FLUS nodules,
rFNA-T2 - the cut-off value set at category III of rFNA outcome for AUS nodules or at subcategory AUS of category III for FLUS nodules,
EU-TIRADS 5 – the cut-off value set at category 5 EU-TIRADS,
EU-TIRADS 4 – the cut-off value set at category 4 EU-TIRADS.

| Measure | rFNA-T1 | | | rFNA-T2 | | | EU-TIRADS 5 | | | EU-TIRADS 4 | | | *rFNA-T1 vs EU-TIR 5 p* | | *rFNA-T1 vs EU-TIRADS 4 p* | | *rFNA-T2 vs EU-TIRADS 5 p* | | *rFNA-T2 vs EU-TIRADS 4 p* | |
|---|---|---|---|---|---|---|---|---|---|---|---|---|---|---|---|---|---|---|---|---|
| | AUS | FLUS | *p* | AUS | FLUS | *p* | AUS | FLUS | *p* | AUS | FLUS | *p* | AUS | FLUS | AUS | FLUS | AUS | FLUS | AUS | FLUS |
| TP | 5/21.7 | 9/6.0 | ***0.0096*** | 10/43.5 | 13/8.6 | ***<0.0000*** | 6/26.1 | 12/7.9 | ***0.0078*** | 11/47.8 | 22/14.6 | ***0.0002*** | 0.7296 | *0.4973* | ***0.0633*** | ***0.0137*** | *0.2156* | *0.8346* | *0.7672* | *0.1057* |
| TN | 10/43.5 | 123/81.5 | ***0.0001*** | 3/13.0 | 110/72.8 | ***<0.0000*** | 10/43.5 | 121/80.1 | ***0.0001*** | 7/30.4 | 60/39.7 | *0.3932* | 1.0 | *0.7702* | *0.3595* | ***<0.0001*** | ***0.0495*** | *0.1355* | *0.2836* | ***<0.0001*** |
| FP | 0/0.0 | 2/1.3 | *0.6207* | 7/30.4 | 15/9.9 | ***0.0059*** | 0 | 4/2.6 | *0.9658* | 3/13.0 | 65/43.0 | ***0.0118*** | - | *0.6801* | *0.2324* | ***<0.0001*** | ***0.0138*** | ***0.0178*** | *0.2836* | ***<0.0001*** |
| FN | 8/34.8 | 17/11.3 | *0.0027* | 3/13.0 | 13/8.6 | *0.7655* | 7/30.4 | 14/9.3 | ***0.0037*** | 2/8.7 | 4/2.6 | *0.3859* | 0.7531 | *0.5695* | *0.0739* | ***0.0066*** | *0.2836* | *0.8402* | *0.6357* | ***0.0458*** |
| SEN | 38.5 | 34.6 | *0.8134* | 76.9 | 50.0 | *0.2055* | 46.2 | 46.2 | *0.7333* | 84.6 | 84.6 | *1.0* | 0.6914 | *0.3965* | ***0.0439*** | ***0.0007*** | *0.2265* | *0.7814* | *0.6188* | ***0.0180*** |
| SPC | 100.0 | 98.4 | *0.3385* | 30.0 | 88.0 | ***<0.0000*** | 100.0 | 96.8 | *0.6930* | 70.0 | 48.0 | *0.3124* | - | *0.6794* | *0.2104* | ***<0.0001*** | ***0.0049*** | ***0.0170*** | *0.1797* | ***<0.0001*** |
| ACC | 65.2 | 87.4 | ***0.0062*** | 56.5 | 81.5 | ***0.0070*** | 69.6 | 88.1 | ***0.0184*** | 78.3 | 54.3 | ***0.0304*** | 0.7531 | *0.8607* | *0.3259* | ***<0.0001*** | *0.3595* | *0.1093* | *0.5550* | ***<0.0001*** |
| PPV | 100.0 | 81.8 | *0.8385* | 58.8 | 46.4 | *0.4200* | 100.0 | 75.0 | *0.4633* | 78.6 | 25.3 | ***0.0003*** | - | *0.9583* | *0.6792* | ***0.0006*** | *0.1712* | *0.1275* | *0.4328* | ***0.0345*** |
| NPV | 55.6 | 87.9 | ***0.0004*** | 50.0 | 89.4 | ***0.0259*** | 58.8 | 89.6 | ***0.0005*** | 77.8 | 93.8 | *0.3244* | 0.8452 | *0.6422* | *0.4811* | *0.2998* | *0.9171* | *0.9585* | *0.5762* | *0.4797* |
| No/% of nodules | 5/21.7 | 11/7.3 | ***0.0254*** | 17/73.9 | 28/18.5 | ***<0.0001*** | 6/26.1 | 16/10.6 | ***0.0373*** | 14/60.9 | 87/57.6 | *0.7683* | 0.7296 | *0.3133* | ***0.0070*** | ***<0.0001*** | ***0.0012*** | *0.0503* | *0.3454* | ***<0.0001*** |

**Table S6.** Relation between category of EU-TIRADS and the outcome of rFNA for excised AUS and FLUS nodules. Number of cancers shown in parentheses.

| **Categories of EU-TIRADS** | **Categories of rFNA** | | | | | | |
|---|---|---|---|---|---|---|---|
| | **I** | **II** | **III** | **IV** | **V** | **VI** | **all** |
| **AUS nodules** | | | | | | | |
| EU-TIRADS 2 | 0 | 0 | 0 | 0 | 0 | 0 | 0 |
| EU-TIRADS 3 | 0 | 2 | 6 (1) | 0 | 1 (1) | 0 | 9 (2) |
| EU-TIRADS 4 | 0 | 2 (1) | 4 (2) | 0 | 1 (1) | 1 (1) | 8 (5) |
| EU-TIRADS 5 | 1 (1) | 1 (1) | 2 (2) | 0 | 1 (1) | 1 (1) | 6 (6) |
| all | 1 (1) | 5 (2) | 12 (5) | 0 | 3 (3) | 2 (2) | 23 (13) |
| **FLUS nodules** | | | | | | | |
| EU-TIRADS 2 | 0 | 0 | 0 | 0 | 0 | 0 | 0 |
| EU-TIRADS 3 | 6 | 27 | 28 (3) | 2 | 1 (1) | 0 | 64 (4) |
| EU-TIRADS 4 | 7 (1) | 15 (2) | 38 (5) | 7 | 3 (2) | 1 | 71 (10) |
| EU-TIRADS 5 | 0 | 0 | 9 (5) | 1 (1) | 3 (3) | 3 (3) | 16 (12) |
| all | 13 (1) | 42 (2) | 75 (13) | 10 (1) | 7 (6) | 4 (3) | 151 (26) |

**Figure S1.** Distribution of surgically treated nodules regarding their initial diagnosis (FLUS vs. AUS).

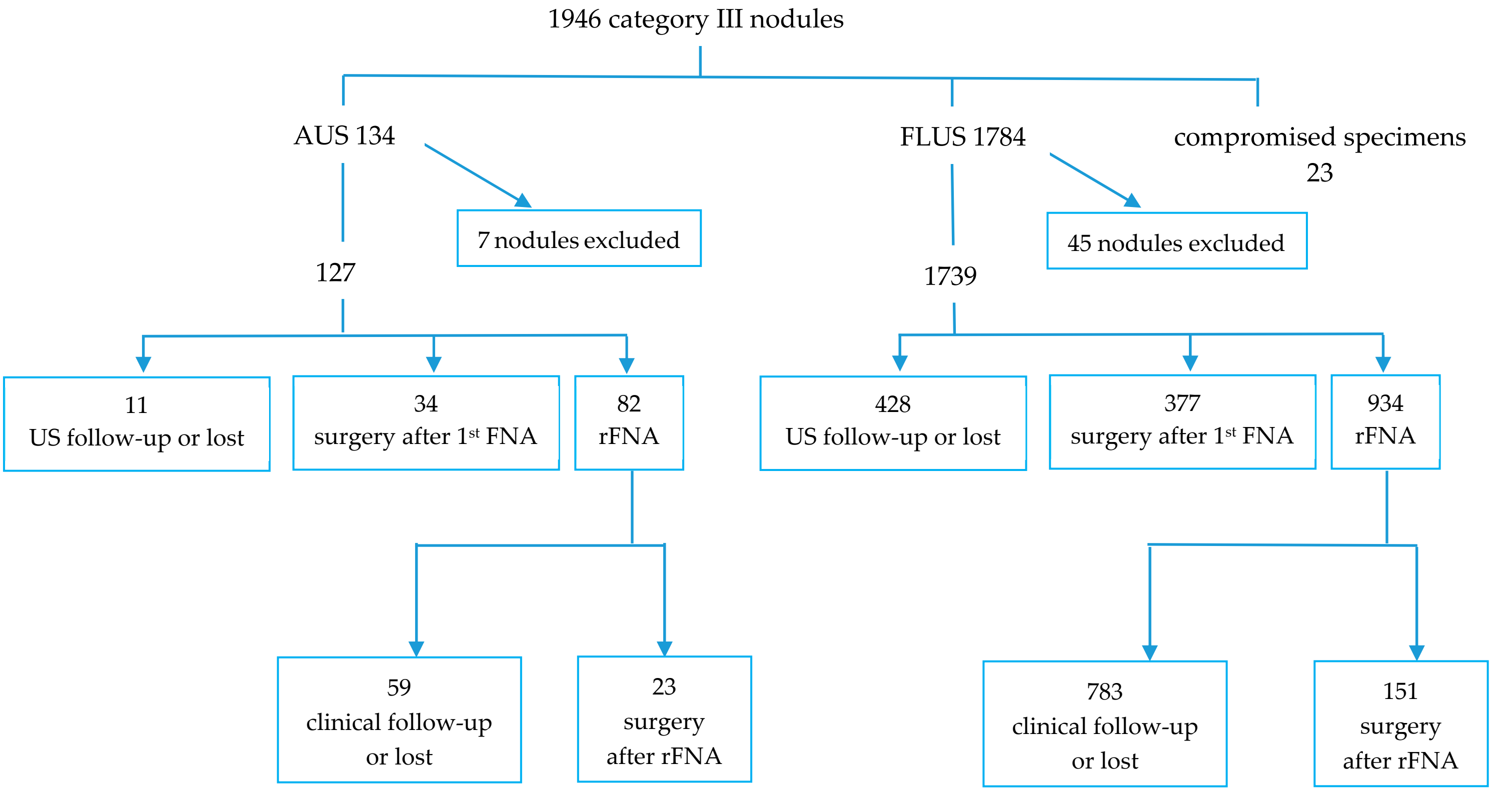

**Figure S2.** ROC curve analysis of the evaluation of diagnostic value of EU-TIRADS and rFNA categories in AUS and FLUS nodules; points of maximal ACC indicated on all curves

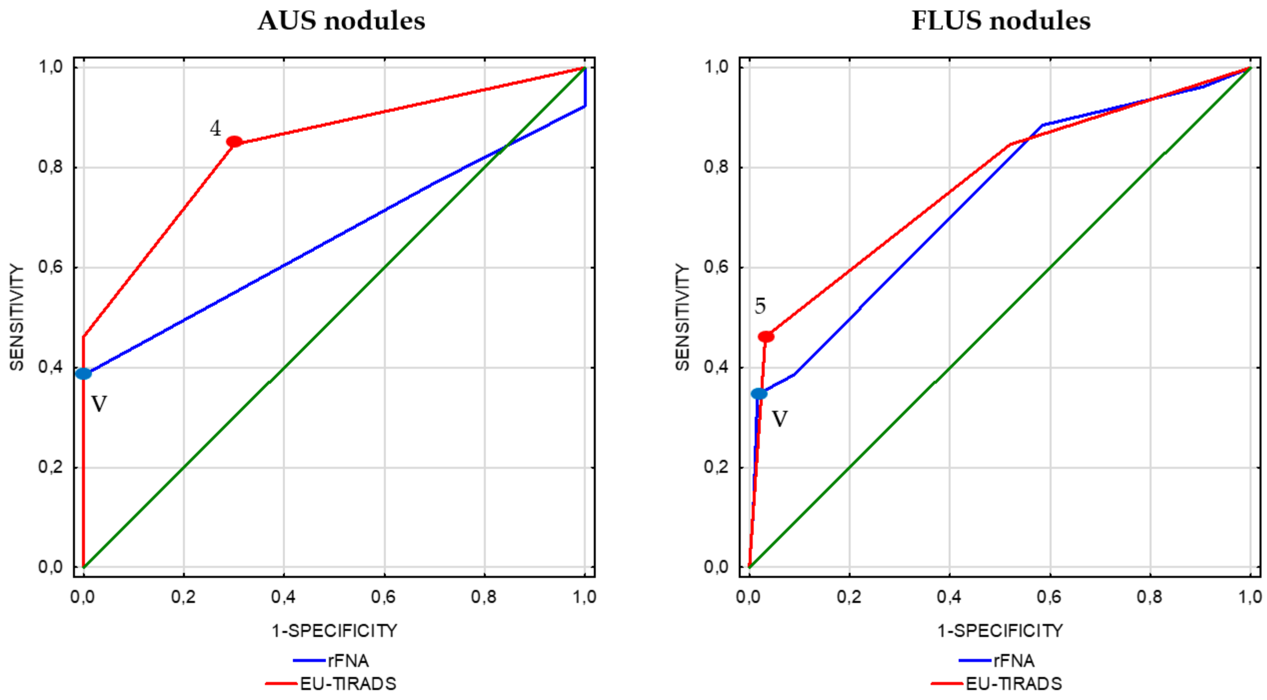

**Figure S3.** Suggested diagnostic algorithms for both types of nodules of category III (as diagnosed in first FNA)

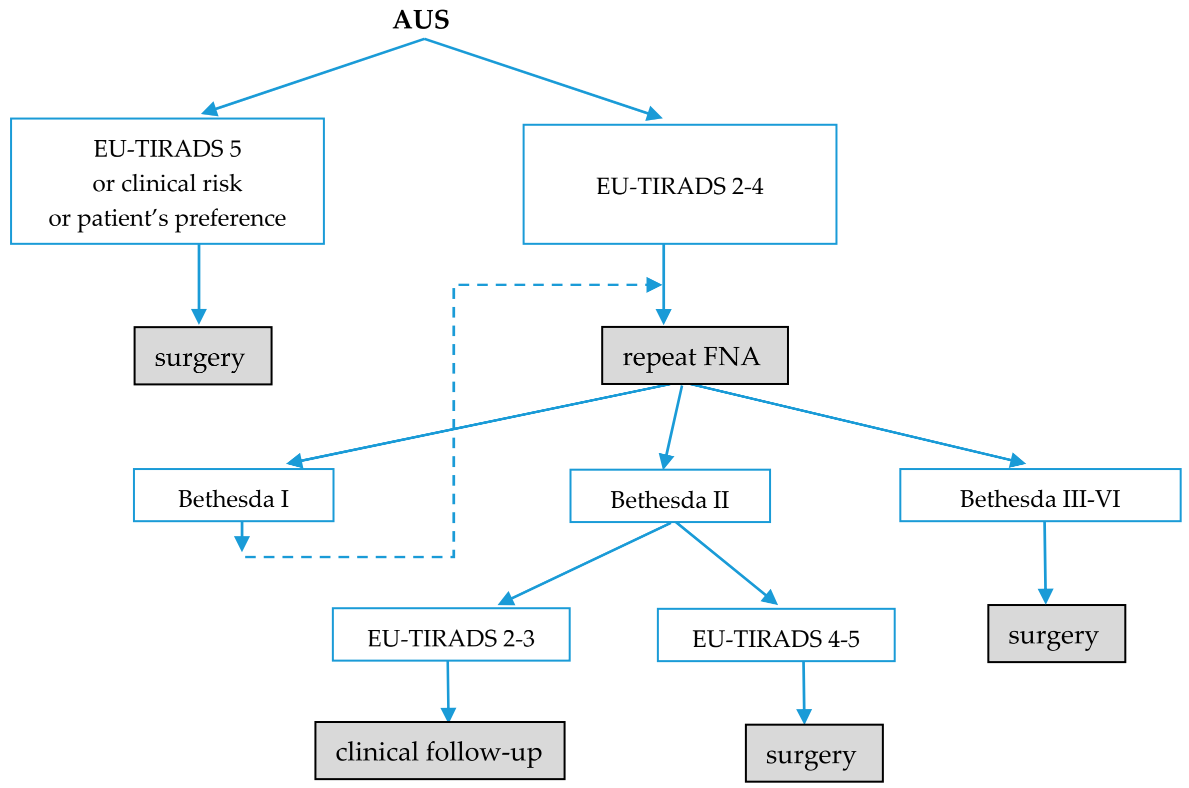


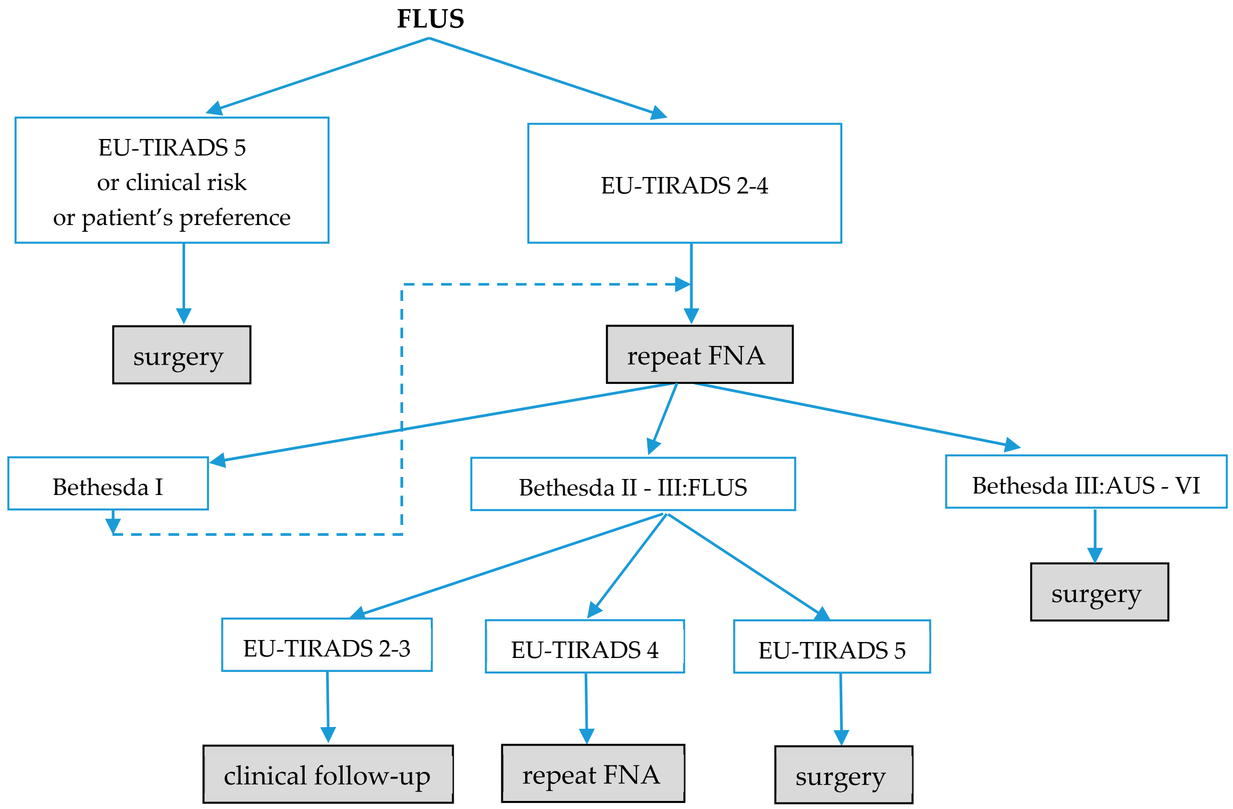


Note: surgery may be replaced with active surveillance or thermal ablation for small nodules, especially in the case of nodules with papillary thyroid carcinoma suspected or diagnosed in repeat FNA, in patients without features of lymph node or distant metastases nor evidence of extrathyroidal extension – if local recommendations allow such an approach
